# Supplementary material for: Lamellar architectures in stiff biomaterials may not always be templates for enhancing toughness in composites
Source: Nat Commun. 2020 Jan 17;11:373. doi: 10.1038/s41467-019-14128-8 (PMC6969223; doi:10.1038/s41467-019-14128-8)
Supplement: Supplementary file 1 — Supplementary Information [file 41467_2019_14128_MOESM1_ESM.pdf]

Supplementary Information

Lamellar Architectures in Stiff Biomaterials May Not Always Be Templates for Enhancing Toughness in Composites

Monn et al.

Supplementary Note 1

The following is a derivation of the equation for fracture initiation toughness used in Section *Measurements of fracture initiation toughness*.

The energy release rate at an applied displacement w_s and crack length Δa is given by

$$G(\Delta a; w_\mathrm{s}) = -\frac{d\Pi(\Delta a; w_\mathrm{s})}{d\Delta A}, \tag{1}$$

where ΔA is the area of the crack. For a specimen with a circular cross-section containing a notch of length a and crack of length Δa, the intact area of the specimen's cross-section $A(\Delta a)$ can be computed using Equation (8). The area of the crack is then given by

$$\Delta A(\Delta a) = A(0) - A(\Delta a). \tag{2}$$

Consequently, we can rewrite Supplementary Equation (1) as

$$\begin{aligned} G(\Delta a; w_\mathrm{s}) &= -\frac{d\Pi(\Delta a; w_\mathrm{s})}{d\Delta a}\left(\frac{d\Delta A(\Delta a)}{d\Delta a}\right)^{-1} \\ &= -\frac{1}{2\sqrt{(a+\Delta a)(D-(a+\Delta a))}}\frac{d\Pi(\Delta a; w_\mathrm{s})}{d\Delta a}, \end{aligned} \tag{3}$$

Per Irwin's analysis of Griffith's theory of fracture, the necessary condition for the extension of a crack is

$$G(\Delta a; w_\mathrm{s}) \geq R(\Delta a), \tag{4}$$

where $R(\Delta a)$ is the material's crack growth resistance at a crack length Δa.

By taking the equality sign in Supplementary Equation (4) and evaluating it for $\Delta a \to 0$, the fracture initiation toughness is given by

$$R(0) = -\frac{1}{2\sqrt{a(D-a)}}\left.\frac{d\Pi(\Delta a; w_\mathrm{s})}{d\Delta a}\right|_{\Delta a=0}, \tag{5}$$

which is identical to Equation (2).

Supplementary Note 2

The following section provides a detailed description of the computational mechanics model used to compute fracture initiation toughness in Section *Measurements of fracture initiation toughness*.

We describe the geometry of the model using the orthonormal set of Cartesian basis vectors $\{\hat{\mathbf{e}}_1, \hat{\mathbf{e}}_2, \hat{\mathbf{e}}_3\}$, shown in Supplementary Figure 2 (A), and their corresponding Cartesian coordinates $\{x_1, x_2, x_3\}$. The origin of this coordinate system is $\mathscr{O}$.

The *Ea.* spicules have a relatively constant diameter. Therefore, we modeled each of them as a cylinder of length L and cross-sectional diameter D. To reduce computational cost, we modeled one quarter of each specimen for which $x_1 \in [0, L/2]$ and $x_3 \leq 0$ (see Supplementary Figure 2 (A)). Thus, the domain of the model for an *Ea.* spicule is

$$\mathscr{B} = \left\{ (x_1, x_2, x_3) \in \mathbb{R}^3 \ : \ 0 \leq x_1 \leq \frac{L}{2},\ x_3 \leq 0,\ x_2^2 + x_3^2 \leq \frac{D^2}{4} \right\}.$$

The *Ta.* spicules, on the other hand, are tapered along their length. It has been shown that their shape is well described by the profile of the Clausen column [1]. However, in order to facilitate preparing the CAD model for our computational analysis, we modeled the *Ta.* spicules' shapes as ellipsoids, which is a close approximation to the Clausen column's shape. In each experiment, we assumed that the spicule's geometry was symmetric about the cross-section located beneath the wedge. Consequently, we took the diameter that we measured, D, to be the ellipsoid's minor axis. We did not measure the length (major axis), L_s, of each *Ta.* spicule. However, it has been shown that the *Ta.* spicule's aspect ratio $\lambda = L_s/D$ is relatively constant with a mean value of 53.6 and a standard deviation of 8.7 (N=31) [1]. Therefore, for all of the *Ta.* spicules that we mechanically tested, we assumed that λ=53.6. Again, we modeled one quarter of the specimen to reduce computational cost and therefore the domain of the model for a *Ta.* spicule is

$$\mathscr{B} = \left\{ (x_1, x_2, x_3) \in \mathbb{R}^3 \ : \ 0 \leq x_1 \leq \frac{L}{2},\ x_3 \leq 0,\ \frac{(x_1 - L/2)^2}{\lambda^2} + x_2^2 + x_3^2 \leq \frac{D^2}{4} \right\}.$$

We denote the components of the displacement vector, $\boldsymbol{u}$, in the $\hat{\mathbf{e}}_i$ basis as u_i, $i \in \{1,2,3\}$. We did not include the notch in the geometry of the model explicitly. Rather, we model the effect of a notch of length a and a crack of length Δa by setting $u_1 = 0$ on $\partial\mathscr{B}_1$ (see Supplementary Figure 2 (B)), where

$$\partial\mathscr{B}_1 = \left\{(x_1,x_2,x_3) \in \mathscr{B} \,:\, x_1 = \frac{L}{2},\, x_2 \geq a + \Delta a - \frac{D}{2}\right\}. \tag{6}$$

This leaves the region of $\mathscr{B}$ for which $x_1 = L/2$ and $x_2 < a + \Delta a - D/2$ traction free, as would be the case for the faces of a notch or crack. We measured the notch length a for each spicule that we mechanically tested from micrographs taken during the FIB notching procedure (see Section *Results*). The values of a are shown in Supplementary Tables 2 and 3.

The other boundary conditions in our model (see Supplementary Figure 2 (A), (B)) are

$$u_1 = u_2 = u_3 = 0 \qquad \text{on} \quad \partial\mathscr{B}_2, \tag{7}$$

$$u_3 = 0 \qquad \text{on} \quad \partial\mathscr{B}_3, \tag{8}$$

$$u_2 = -w_{\text{sim}} \qquad \text{on} \quad \partial\mathscr{B}_4, \tag{9}$$

where w_{sim} is the applied displacement and

$$\partial\mathscr{B}_2 = \{(x_1,x_2,x_3) \in \mathscr{B} \,:\, x_1 = 0\}, \tag{10}$$

$$\partial\mathscr{B}_3 = \{(x_1,x_2,x_3) \in \mathscr{B} \,:\, x_3 = 0\}, \tag{11}$$

$$\partial\mathscr{B}_4 = \left\{(x_1,x_2,x_3) \in \mathscr{B} \,:\, x_1 = \frac{L}{2},\, x_2^2 + x_3^2 = \frac{D^2}{4},\, x_2 > 0.9\frac{D}{2}\right\}. \tag{12}$$

Before crack growth initiation, the F–w_0 response of both the *Ea.* and *Ta.* spicules is linear (see e.g., Figure 5 (C), (D)). This suggests that both the *Ea.* and *Ta.* spicules behave in a linear elastic fashion until the onset of crack growth. The *Ta.* spicules do not appear to possess any internal architecture (see Figure 6 (B) and 2 (F)). Based on this observation we assume that they are elastically homogeneous and

therefore model them as homogeneous linear elastic solids such that

$$\sigma_{ij} = \frac{E}{1+\nu}\left(\varepsilon_{ij} + \frac{\nu}{1-2\nu}\delta_{ij}\sum_{k=1}^{3}\varepsilon_{kk}\right), \tag{13}$$

where σ_{ij} and ε_{ij}, $i,\ j \in \{1,2,3\}$ are, respectively, the components of the Cauchy stress tensor, $\boldsymbol{\sigma}$, and the infinitesimal strain tensor, $\boldsymbol{\epsilon}$, in the dyadic basis $\hat{\mathbf{e}}_i \otimes \hat{\mathbf{e}}_j$. The strain components can be computed from the displacements as $\varepsilon_{ij} = (\partial u_i/\partial x_j + \partial u_j/\partial x_i)/2$, $i,\ j \in \{1,2,3\}$. The symbol δ_{ij} is the Kronecker delta, which is equal to unity when $i = j$ and is zero otherwise. The parameters E and ν are the Young's modulus and Poisson's ratio, respectively.

While the *Ea.* spicules are clearly not homogeneous, we model them as homogeneous linear elastic solids as well. Because of this modeling assumption, the values of $R(0)$ that we obtain for the *Ea.* spicules should be considered effective fracture initiation toughness.

For each *Ea.* and *Ta.* spicule that we mechanically tested, we measured D and L from micrographs (see Supplementary Tables 2 and 3) and computed the compliance of the completely failed specimen $C_\mathrm{f} = w_\mathrm{f}/F_\mathrm{f}$ from the F–w_0 data (see Supplementary Tables 2 and 3). A completely failed spicule can be modeled as two cantilevers that share the load equally. In this completely failed state, the effective Young's modulus can be computed using Euler-Bernoulli beam theory as $E = L^3/48C_\mathrm{f}I$, where $I = \pi D^4/64$. We did not measure the Poisson's ratio ν and took its value to be 0.2, which is typical for glass [2].

In equilibrium, the Cauchy momentum equation requires that

$$\mathrm{Div}(\boldsymbol{\sigma}) = 0 \quad \text{on} \quad \mathscr{B}, \tag{14}$$

where $\mathrm{Div}(\cdot)$ is the divergence operator.

We solved Supplementary Equations (13) and (14) subject to the boundary conditions Supplementary Equations (6)–(9) for a given value of Δa using finite element methods [3]. A representative finite element mesh is shown in Supplementary Figure 2 (C). The mesh consists of constant strain tetrahedron elements. Supplementary Figure 3 (A), (B) shows contours of the σ_{11} component of the Cauchy stress obtained

from the finite element calculations.

The experiment described in Section *Results* consists of a specimen with a crack placed in series with a linear spring (see e.g., Figure 5 (A)). We denote the elastic compliance of this spring as C_{m} and the elastic compliance of the specimen as $C(\Delta a)$, where Δa is the length of the crack. By moving the translation stage, we apply a displacement w_s to the spring-specimen system. This consequently results in a displacement of the specimen given by

$$w_0(\Delta a; w_{\mathrm{s}}) = \frac{w_{\mathrm{s}} C(\Delta a)}{C_{\mathrm{m}} + C(\Delta a)}. \tag{15}$$

For a fixed w_{s}, the potential energy of the system is

$$\Pi(\Delta a; w_{\mathrm{s}}) = \frac{w_{\mathrm{s}}^2}{2} \frac{1}{C_{\mathrm{m}} + C(\Delta a)} \tag{16}$$

By substituting Supplementary Equation (15) and Supplementary Equation (16) into Equation (2), the fracture initiation toughness is given by

$$R(0) = \frac{1}{2} \left(\frac{w_{\mathrm{c}}}{C(0)} \right)^2 \frac{1}{2\sqrt{a(D-a)}} \left. \frac{dC(\Delta a)}{d\Delta a} \right|_{\Delta a = 0}, \tag{17}$$

where w_{c} is the value of w_0 at the initiation of crack growth.

In contrast to the experiment described above, our computational mechanics model is displacement controlled and therefore the machine compliance $C_{\mathrm{m}} = 0$. We denote the applied displacement in the simulation as w_{sim} and simplify Supplementary Equation (16) to be

$$\Pi_{\mathrm{sim}}(\Delta a; w_{\mathrm{sim}}) = \frac{w_{\mathrm{sim}}^2}{2C(\Delta a)}. \tag{18}$$

Computing the derivative of Π_{sim} in Supplementary Equation (18) gives us

$$\left. \frac{d\Pi_{\mathrm{sim}}(\Delta a; w_{\mathrm{sim}})}{d\Delta a} \right|_{\Delta a = 0} = -\frac{1}{2} \left(\frac{w_{\mathrm{sim}}}{C(0)} \right)^2 \left. \frac{dC(\Delta a)}{d\Delta a} \right|_{\Delta a = 0}. \tag{19}$$

Finally, combining Supplementary Equation (17) and Supplementary Equation (19) we find that

$$R(0) = -\left(\frac{w_{\mathrm{c}}}{w_{\mathrm{sim}}}\right)^2 \frac{1}{2\sqrt{a(D-a)}} \left.\frac{d\Pi_{\mathrm{sim}}(\Delta a;\, w_{\mathrm{sim}})}{d\Delta a}\right|_{\Delta a=0}. \tag{20}$$

To compute $R(0)$ using Supplementary Equation (20) we must first compute $d\Pi_{\mathrm{sim}}/d\Delta a|_{\Delta a=0}$ from the computational mechanics model described above.

For each spicule that we mechanically tested we generated four models with different crack lengths $\Delta a = nh$, where $h = 0.08D$ and $n \in \{-2,-1,1,2\}$. As we varied the crack length Δa, we kept the applied displacement fixed at w_{sim}. For each model we solved for the equilibrium displacement field using finite element methods [3] as described in the preceding sections. We computed the potential energy of the system as

$$\Pi_{\mathrm{sim}}(\Delta a;\, w_{\mathrm{sim}}) = 4\int_{\mathscr{B}} \frac{E}{2(1+\nu)} \sum_{i=1}^{3}\sum_{j=1}^{3} \left[\frac{1}{2}\left(\frac{\partial u_i}{\partial x_j}\frac{\partial u_i}{\partial x_j} + \frac{\partial u_i}{\partial x_j}\frac{\partial u_j}{\partial x_i}\right) + \frac{\nu}{1-2\nu}\frac{\partial u_i}{\partial x_i}\frac{\partial u_j}{\partial x_j}\right] d\mathscr{B}. \tag{21}$$

The factor of four in front of the integral in Supplementary Equation (21) comes from the fact that our computational mechanics model consists of one quarter of the full specimen. Values of $\Pi_{\mathrm{sim}}(\Delta a;\, w_{\mathrm{sim}})$ for a representative spicule are shown in Supplementary Figure 3 (C). Finally, the derivative $d\Pi_{\mathrm{sim}}/d\Delta a$ in Supplementary Equation (20) was computed numerically using the central finite difference method such that

$$\left.\frac{d\Pi_{\mathrm{sim}}(\Delta a;\, w_{\mathrm{sim}})}{d\Delta a}\right|_{\Delta a=0} = \frac{\Pi_{\mathrm{sim}}(-2h;\, w_{\mathrm{sim}}) - 8\Pi_{\mathrm{sim}}(-h;\, w_{\mathrm{sim}}) + 8\Pi_{\mathrm{sim}}(h;\, w_{\mathrm{sim}}) - \Pi_{\mathrm{sim}}(2h;\, w_{\mathrm{sim}})}{12h} + O(h^4), \tag{22}$$

where O is the Landau symbol big-O.

Using this procedure we computed $R(0)$ from Supplementary Equation (20) for each spicule that we mechanically tested. The values of $R(0)$ are shown in Supplementary Tables 2 and 3.

Supplementary Note 3

Motivated by the work of Ambrosio and Tortorelli [4,5], the variational fracture theory (VFT) was put forward by Francfort and Marigo [6] to model the evolution of cracks in brittle solids.

In it, the solution displacement field, $\mathbf{u}$, and the crack (or system of cracks), Γ, are postulated to be those that globally minimize the system's total potential energy. However, from what is understood and observed about the physics and mechanics of fracture, it is more reasonable to postulate that the experimentally observed $(\mathbf{u},\Gamma)$ are only local minimizers of the total potential energy. There are also several other limitations in VFT about what type of fracture scenarios it can model. Despite the over-constraints on the solution $(\mathbf{u},\Gamma)$ in the VFT and its other limitations, it has been shown that its predictions of crack paths are surprisingly close to experimental observations [7,8]. Considering that, we used the VFT to study the effect of a weak interface's geometry on its ability to enhance the work of fracture (see Section *Discussion*).

It is typically quite challenging to solve for $(\mathbf{u},\Gamma)$ in the VFT, even using numerical methods. In order to obtain a numerical solution, the VFT is regularized (see Bourdin et. al [8] for details) so that the total energy is

$$E_\ell(\mathbf{u},d) = \int_{\mathscr{B}} (1-d)^2 \Psi_0(\mathbf{x},\mathbf{u})\, d\mathscr{B} + \int_{\mathscr{B}} \frac{G_\mathrm{c}}{2} \left(\frac{d(\mathbf{x})^2}{\ell} + \ell\, \|\boldsymbol{\nabla} d(\mathbf{x})\|^2 \right) d\mathscr{B}, \tag{23}$$

The first term on the right hand side of Supplementary Equation (23) is the strain energy stored in the solid, while the second term is the energy of the new surface area created by the growth of the crack. In Supplementary Equation (23), Ψ_0 is the strain energy density, $\mathbf{x}$ is the position vector of a material point in the solid $\mathscr{B}$, and $\mathbf{u}$ is the displacement field. The operator $\|\cdot\|$ is the Euclidean 2-norm, and $\boldsymbol{\nabla}(\cdot)$ is the gradient operator with respect to $\mathbf{x}$. For a brittle material, the crack growth resistance, R is constant and we take its value to be G_c.

The crack is represented by the scalar valued field d, termed the phase field or the damage field, which takes values between zero and unity. The field d indicates the extent of damage in the material such that at $d=1$ the material is fully damaged and at $d=0$ it is completely intact. Therefore, the crack is the set

of points in $\mathscr{B}$ for which d is close to unity. The parameter ℓ is called the regularization parameter. It has been shown that the minimizers of the energy given by Supplementary Equation (23) converge to the minimizers of the energy in the VFT theory as $\ell \to 0$ (see [4,9,10] for details).

While cracks in RVFT are defined to be subsets of $\mathscr{B}$ and therefore have finite thickness, this does not preclude RVFT from being a useful and predictive theory of fracture. However, for the cracks in RVFT to be physically meaningful their thicknesses should be much smaller than the dimensions of the solid. This is because the crack region has zero stiffness. Consequently, if a cracks thickness is not much smaller than the solids dimensions then the failure behavior is more similar to plastic failure than brittle fracture. It is argued that the thickness of the crack is of the order of ℓ[11]. Therefore, by choosing ℓ to be much smaller than the characteristic dimension of the solid one can ensure that the results have the physical features of brittle fracture. However, from a practical perspective, the computational cost grows inversely with ℓ. This leads to what may be the greatest limitation of RVFT—it is computationally expensive. This limits the complexity of the architectures and size of the specimens that can be modeled. This is part of the reason that we only model two layers and their adjoining interface for the planar and cylindrical layered architectures that we discuss in Section *Discussion*.

A necessary condition for $\mathbf{u}$ and d (which is a representation of Γ) to be minimizers of E_ℓ is that they satisfy Supplementary Equation (14), in which $\boldsymbol{\sigma} = (1-d)^2 \partial\Psi_0/\partial\boldsymbol{\epsilon}$, and

$$G_c\, \ell\, \boldsymbol{\nabla}\cdot(\boldsymbol{\nabla} d) - \frac{G_c\, d}{\ell} = -2(1-d)\Psi_0, \qquad \text{on} \quad \mathscr{B}. \tag{24}$$

subject to the boundary conditions

$$\mathbf{u} = \hat{\mathbf{u}}, \qquad \text{on} \quad \partial\mathscr{B}_u, \tag{25a}$$

$$\boldsymbol{\sigma}\mathbf{n} = \mathbf{t}, \qquad \text{on} \quad \partial\mathscr{B}_t, \tag{25b}$$

$$\boldsymbol{\nabla} d\cdot\mathbf{n} = 0, \qquad \text{on} \quad \partial\mathscr{B}, \tag{25c}$$

where $\partial\mathscr{B}$ is the boundary of the solid, $\partial\mathscr{B}_u$ is the part of $\partial\mathscr{B}$ on which displacements $\hat{\mathbf{u}}$ are prescribed, and $\partial\mathscr{B}_t$ is the rest of the boundary on which tractions $\mathbf{t}$ are prescribed. The symbol $\mathbf{n}$ is the outward

normal vector on $\partial\mathscr{B}$.

We solved Supplementary Equation (14) and (24), subject to the boundary conditions given by Supplementary Equation (25) using the finite element-based procedure detailed in[12]. The results from this calculation are shown in Figure 9. We have also carried out simulations to experimentally validate our finite element implementation of RVFT. The results from those simulations and similar results reported in literature (e.g. see [13–15]) show that RVFT is a dependable tool for gaining qualitative insight into fracture mechanics phenomena. There are, however, several issues that prevent RVFT from being used to make quantitative predictions. For example, crack growth in RVFT can occur in the presence of dominant local compressive stresses. This phenomenon is clearly not physical. One of the most used strategies to circumvent this problem is Miehe et al.'s tension-compression split[14]. In this strategy, d affects only a part of Ψ_0 that is composed of the positive eigenvalues of the strain tensor in Supplementary Equation (23) and Supplementary Equation (24). While this strategy and several others have been proposed[16–18], the resolution of the compressive failure problem remains an active area of research. We have ensured that the compressive failure problem and other related issues that are known to limit the RVFT's predictive capability were negligible in our simulations.

In this work, we modified the regularized VFT (RVFT) proposed by[8] to allow us to model interfaces with different fracture toughnesses within a solid. We did so by spatially varying G_c such that $G_\mathrm{c} = G_\mathrm{c}(\mathbf{x})$. However, the difference between the dimensionality of an interface (a subset of $\mathbb{R}^2$) and a solid (a subset of $\mathbb{R}^3$) results in the same problem that arises when modeling cracks in the VFT. We overcame this problem using a similar regularization strategy as was used in RVFT. Namely, we replaced the interface with a thin interfacial region of thickness $m\ell$, where $m \in \mathbb{R} > 0$ is a fixed constant, so that as $\ell \to 0$ the interfacial region becomes vanishingly thin. We chose $G_\mathrm{c}(\mathbf{x})$ to be a piecewise continuous function so that $G_\mathrm{c} = G_\mathrm{I}$ in the interfacial region, and $G_\mathrm{c} = G_\mathrm{b}$ in the bulk material. In the simulations whose results we present in Section *Discussion* we take $m = 2$ and $\ell = 0.05$ mm.

To reduce the computational cost of our virtual experiments, we assumed that the crack path was symmetric about $x_1 = L/2$ and $x_3 = 0$ (see Figure 9). Therefore, we modeled one quarter of the geometry for which $x_1 \in [0, L/2]$ and $x_3 \leq 0$ and imposed the boundary conditions $u_1 = 0$ at $x_1 = L/2$ and $u_3 = 0$ at $x_3 = 0$.

Supplementary Methods

We performed our fracture tests in air on sections of *Ea.* spicules obtained from dry *Ea.* skeletons. However, in its native state the *Ea.* sponge lives anchored to the sea floor. It has been shown that the mechanical behaviors of some biological materials (such as nacre [19], antler [20] and bone [20]) change drastically if they are dried out prior to mechanical testing. For example, the work of fracture of nacre that has been soaked in water is almost triple that of nacre that is stored in dry conditions [21].

To determine whether storing the *Ea.* spicules in dry conditions had a significant effect on their mechanical behavior, we soaked sections of 11 *Ea.* spicules in artificial seawater for 16 days and then performed three-point bending tests on them using the procedure described in [22].

After soaking an *Ea.* spicule section in artificial seawater, we placed it across a trench cut in a cut in a steel plate. The trench span, L, was measured from optical micrographs to be 1.278 mm. We used the same mechanical testing device described in Section *Results* and in [22] to load the spicule sections in three-point bending and measure F and w_0 until they failed. However, unlike the experiments described in Section *Results* we did not notch the spicule sections nor did we glue their ends to the steel plate before testing them. After each test we mounted the pieces of the broken spicule to a aluminum stub, coated them in 10 nm of carbon and imaged them in a scanning electron microscope (SEM). We measured each spicule's diameter, D, at the cross-section where it failed from SEM images.

We used the slope k_i of the initial linear portion of the spicule's F-w_0 response to compute the Young's moduli of both the 11 wet spicules and the 33 dry spicules whose F-w_0 responses were previously reported in [22]. We computed the Young's moduli using Euler-Bernoulli beam theory [23] as

$$E = \frac{k_\mathrm{i} L^3}{48 I}, \tag{26}$$

where $I = \pi D^4/64$. A histogram of the Young's moduli of the wet and dry spicules is shown in Figure Supplementary Figure 1 (A).

The bending failure strains, ε_f, of the dry spicules have already been reported in [22]. We computed the bending failure strain, ε_f, of the wet spicules using the same procedure, which is described in Section 2

of[22]. We selected points along the spicule's longitudinal axis from a micrograph of the spicule taken during the bending test just before the spicule failed. We fit a polynomial to these points and used it to compute the curvature of the spicule's longitudinal axis. Finally, we computed ε_{f} using the maximum value of the curvature, κ^*, as $\varepsilon_{\mathrm{f}} = D\kappa^*/2$. We compared ε_{f} for the wet spicules to ε_{f} for the dry spicules in Supplementary Figure 1 (B).

From these experiments, we do not see any difference in the Young's modulus or bending failure strain between the dry spicules and the spicules that were soaked in seawater. Bias-corrected accelerated (BCa) confidence intervals (CI) using 10,000 bootstrapped samples indicated no reliable difference in the means of the Young's moduli of the wet and dry spicules (upper: 3.0 GPa; lower: -4.5 GPa). Furthermore, a two-sided t-test for independent samples (equal variances not assumed) also indicated no significant difference in their means (degrees of freedom=12, t=0.05, p=0.96). Similar results were found when comparing the bending failure strains using both BCa CI (upper: 0.0012; lower: -0.0034) and the t-test (degrees of freedom=12, t=0.97, p=0.35). From these results we conclude that soaking the spicules in water prior to testing them does not appear to affect their Young's modulus and bending failure strain. Based on these results, we speculate that the toughness properties of the spicules also would not change after being soaked in water.

Supplementary Figures

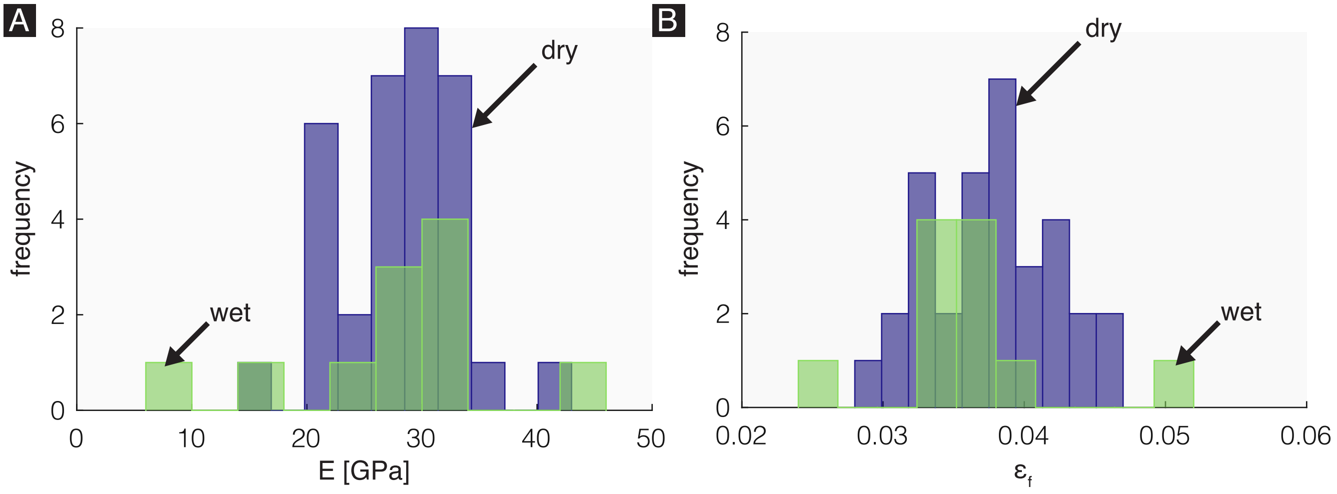

Supplementary Figure 1. Effect of moisture on the mechanical properties of *Ea.* spicules. (A) A histogram of the effective Young's modulus, E, for 33 dry *Ea.* (light blue) and 11 wet *Ea.* (green) spicules. The mean±standard error of E for the dry and wet spicules is 28.1±0.9 GPa and 28.0±2.9 Gpa, respectively. (B) A histogram of the bending failure strain, ε_f, for 33 dry *Ea.* (light blue) and 11 wet *Ea.* (green) spicules. The mean±standard error of ε_f for the dry and wet spicules is 0.0377±0.0008 and 0.0358±0.0019, respectively.

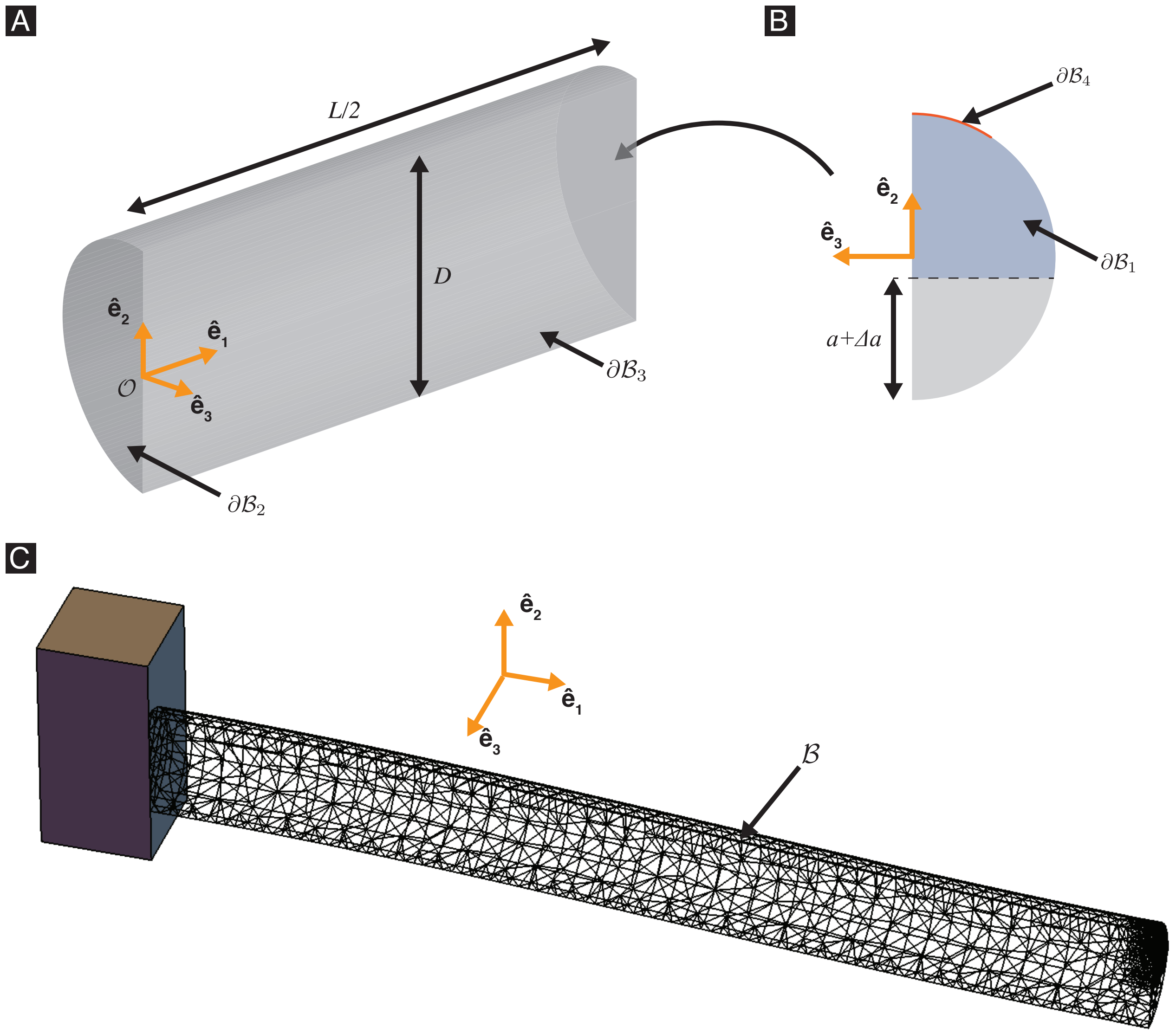

Supplementary Figure 2. Computational mechanics model of the spicules. (A) Geometry of the *Ea.* model consisting of one quarter of the specimen. The Cartesian basis vectors and origin of the Cartesian coordinate system $\mathscr{O}$ are shown. (B) A view of the model's cross section at $x_1 = L/2$ showing the boundaries $\partial\mathscr{B}_1$ and $\partial\mathscr{B}_4$. (C) The deformed finite element mesh of a representative *Ea.* model.

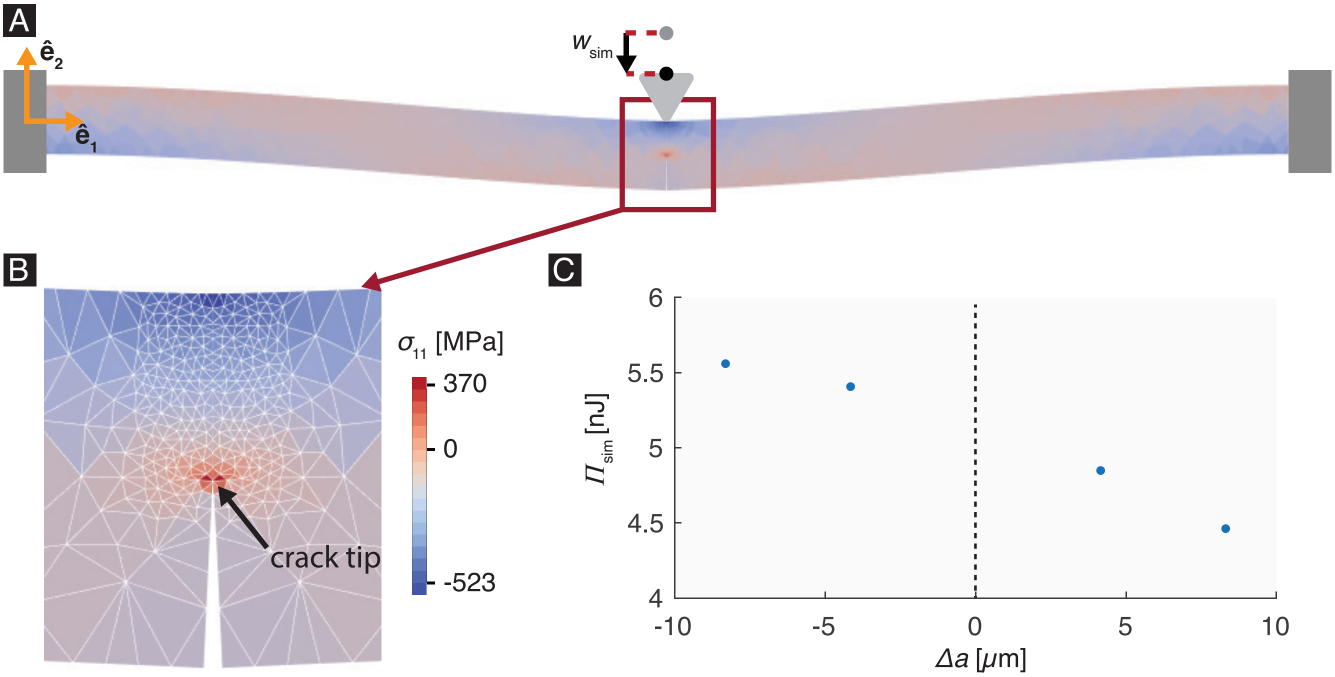

Supplementary Figure 3. Results of the computational mechanics model. (A) Contours of σ_{11} on the surface $\partial\mathscr{B}_3$ (see Supplementary Figure 2 (A)) shown in the deformed configuration for a representative *Ea.* model. The displacements are scaled by a factor of 4. (B) A magnified view of the σ_{11} contours near $x_1 = L/2$. The largest tensile stress (red) occurs near the tip of the crack. (C) The values of Π_{sim} for different Δa obtained from the computational mechanics model for a representative *Ea.* spicule. This data is used to compute the spicule's fracture initiation toughness in Section *Measurements of fracture initiation toughness*. We did not compute the value of Π at $\Delta a = 0$ since it is not required for computing the energy release rate (see Supplementary Equation (22)).

Supplementary Tables

Supplementary Table 1. Summary of SBM crack growth resistance data used to compute the toughness metrics in Figure 8. Statistical data, such as standard deviation (s.d.) standard error (s.e.) and sample number (N) are included when available.

Material	$R(0)$ [Jm^{-2}]	$\langle R \rangle$ [Jm^{-2}] [c)]	Ref.[g)]
nacre (*Pinctada margaritifera*)	587±189 (mean±s.d., N=14)	2068±544 (mean±s.d., N=12)	11
nacre (*Pinctada margaritifera*)	—	3300±78 (mean±s.e.)[d)]	12
nacre (*Pinctada margaritifera*)	—	2880±260 (mean±s.d.)[d)]	7
bone (*Homo sapiens*)	50–200	11250[e)]	35
bone (*Homo sapiens*)	224±2 (mean±s.d., N=7)[f)]	—	28
bone (*Papio anubis*)	—	15520±2900 (mean±s.d., N=3)	36
bone[a)]	—	8000±524 (mean±s.e.)[d)]	12
antler (*Cervus canadensis*)	100[e)]	14000[e)]	29
conch (*Strombus gigas*)	25±7 (mean±s.d., N=4)	26000±14000 (mean±s.d., N=8)	57
calcite[b)]	≈3	—	68
limestone	—	22	69
hydroxyapatite[b)]	≈10	—	68
hydroxyapatite	—	140	70

a) The authors of this work did not specify the genus and species from which the bone specimens were obtained;
b) Approximate values are given because the data was taken from a graph showing ranges of property values;
c) Values were obtained for the work of fracture, γ_{WOF}, and were used to compute $\langle R \rangle = 2\gamma_{\mathrm{WOF}}$;
d) The number of specimens was not given in the referenced work;
e) Values estimated from R-curve data;
f) The reference provides the mode I stress intensity factor, K_I, at crack growth initiation. From this we estimated $R(0)$ to be K_I^2/E, where E is the Young's modulus. We take the value of E to be 18.6 GPa as per [24].
g) The numbering in this column refers to the reference numbering in the main text.

Supplementary Table 2. *Ea.* spicule data. L is the length; D is the diameter of the cross-section midway along the length; a is the notch length; r_n is the notch root radius; $\alpha = a/D$ is the dimensionless notch length; $(w_c,\ F_c)$ and $(w_f,\ F_f)$ are the (w_0, F) points at pop-in and complete failure, respectively; $R(0)$ is the fracture initiation toughness; and $\langle R \rangle$ is the average crack growth resistance.

Specimen ID	L [μm]	D [μm]	a [μm]	r_n [nm]	α	w_c [μm]	F_c [mN]	w_f [μm]	F_f [mN]	$R(0)$ [Jm^{-2}]	$\langle R \rangle$ [Jm^{-2}]
Ea1	604.8	50.75	16.13	25	0.32	3.6	13.37	45.7	52.08	7.62	241.96
Ea2	813.0	37.44	10.83	84	0.29	6.9	4.54	42.3	9.40	7.94	75.35
Ea3	814.6	36.57	9.19	251[a)]	0.25	35.8	24.02	40.1	9.04	—	—[c)]
Ea4	820.5	23.46	4.31	24	0.18	9.9	1.23	76.1	2.60	2.77	—[c)]
Ea5	813.0	25.14	10.13	36	0.40	8.6	1.07	27.7	1.01	6.28	28.28
Ea6	814.6	21.60	13.81	77	0.64	8.8	0.54	28.0	0.72	9.30	23.32
Ea7	820.5	47.07	19.05	300[a)]	0.40	1.7	8.26	38.2	27.95	—	118.68
Ea8	813.0	46.79	14.90	372[a)]	0.32	2.5	11.87	41.0	28.07	—	169.99
Ea9	808.8	46.50	14.92	120	0.32	2.3	7.60	47.6	28.91	1.66	101.20
Ea10	808.8	27.74	7.60	229[a)]	0.27	28.3	9.81	39.9	3.44	—	—[c)]
Ea11	813.0	32.81	15.82	39	0.48	3.7	1.41	28.6	3.53	2.61	25.59
Ea12	820.5	31.33	17.09	69	0.55	—[b)]	—	33.0	2.86	—	63.25
Ea13	783.3	22.94	7.22	39	0.31	4.7	0.81	21.7	0.88	1.62	—[c)]
Ea14	814.6	45.65	22.29	45	0.49	5.9	6.51	49.1	17.73	10.76	136.12
Ea15	814.6	45.51	21.62	84	0.47	—[b)]	—	38.8	15.89	—	118.86
Ea16	808.8	45.59	21.34	79	0.47	—[b)]	—	34.6	14.80	—	—[c)]
Ea17	775.2	51.97	14.86	90	0.29	2.1	4.89	72.8	54.02	1.44	141.87
Ea18	783.3	57.76	18.15	178	0.31	2.1	7.33	52.5	53.60	1.86	170.28
Ea19	790.5	62.61	18.55	84	0.30	2.3	12.01	63.2	76.14	1.94	238.54
Ea20	820.5	64.40	23.36	89	0.36	1.9	14.19	97.6	153.63	1.91	383.81
Ea21	814.6	70.42	24.44	44	0.35	2.6	20.28	68.5	139.05	3.76	335.33
Ea22	779.6	43.16	17.26	52	0.40	1.7	2.54	30.7	15.16	1.11	—[c)]
Ea23	813.0	50.83	19.82	118	0.39	2.6	7.58	82.9	49.56	2.15	278.43
Ea24	814.6	53.12	14.98	57	0.28	2.9	8.95	67.0	48.87	2.59	132.91
Ea25	775.2	57.24	32.40	112	0.57	—[b)]	—	46.8	55.94	—	258.58
Ea26	814.6	24.56	3.07	137	0.13	—[b)]	—	103.0	2.19	—	—[c)]
Ea27	813.0	44.17	2.97	37	0.07	42.5	59.37	121.9	13.99	33.85	—[c)]
Ea28	820.5	24.06	2.96	186	0.12	17.9	2.66	22.0	0.81	7.49	—[c)]
Ea29	820.5	33.13	3.94	43	0.12	—[b)]	—	86.4	10.69	—	—[c)]
Ea30	775.2	40.21	6.24	51	0.16	—[b)]	—	54.0	18.54	—	—[c)]
Ea31	783.3	35.42	6.68	193	0.19	4.1	4.48	62.2	14.09	2.03	—[c)]
Ea32	820.5	39.29	3.06	159	0.08	46.3	77.24	83.6	4.76	27.20	—[c)]
Ea33	814.6	29.51	3.64	136	0.12	—[b)]	—	69.3	4.56	—	—[c)]
Ea34	775.2	46.92	4.41	153	0.09	25.7	78.30	94.1	12.64	19.46	—[c)]
Ea35	783.3	34.02	5.07	136	0.15	—[b)]	—	84.8	12.43	—	—[c)]

[a)] The value of r_n exceeds the threshold for valid measurement of $R(0)$ as per Section *Measurements of fracture initiation toughness*;

[b)] Values of w_c and F_c could not be obtained for this specimen since we could not reliably identify the pop-in event.

[c)] In this specimen, the value of ΔF_c exceeded 15% of the value of F_c and therefore we did not consider crack growth to be predominantly stable.

Supplementary Table 3. *Ta.* spicule data. L is the length; D is the diameter of the cross-section midway along the length; a is the notch length; r_n is the notch root radius; $\alpha = a/D$ is the dimensionless notch length; $(w_c,\ F_c)$ and $(w_f,\ F_f)$ are the (w_0, F) points at pop-in and complete failure, respectively; $R(0)$ is the fracture initiation toughness; and $\langle R \rangle$ is the average crack growth resistance.

Specimen ID	L [μm]	D [μm]	a [μm]	r_n [nm]	α	w_c [μm]	F_c [mN]	w_f [μm]	F_f [mN]	$R(0)$ [Jm^{-2}]	$\langle R \rangle$ [Jm^{-2}]
Ta1	775.2	23.67	5.72	107	0.24	4.4	0.93	13.7	0.43	0.81	—[c)]
Ta2	783.3	28.00	13.06	365[a)]	0.47	8.9	1.60	25.1	2.09	—	—[c)]
Ta3	790.5	30.34	12.71	129	0.42	6.6	0.60	27.1	0.66	1.66	—[c)]
Ta4	601.3	43.61	11.17	63	0.26	3.1	8.66	41.2	23.64	3.93	130.89
Ta5	601.3	36.17	15.92	50	0.44	4.7	4.26	34.0	10.03	10.46	—[c)]
Ta6	597.2	34.69	8.29	35	0.24	3.1	3.46	40.6	12.77	3.16	—[c)]
Ta7	604.8	24.05	8.69	79	0.36	2.6	0.86	20.3	1.88	1.46	19.55
Ta8	601.3	27.84	10.82	87	0.39	4.1	1.49	15.8	2.25	5.47	—[c)]
Ta9	603.8	31.13	10.67	28	0.34	2.1	1.70	24.1	5.89	1.62	—[c)]
Ta10	597.2	37.37	9.37	82	0.25	3.2	5.10	31.0	11.79	3.70	69.94
Ta11	604.8	37.56	14.23	127	0.38	2.9	3.83	34.5	14.94	4.82	84.65
Ta12	597.2	34.45	7.85	216[a)]	0.23	3.9	3.94	27.1	10.92	—	29.91
Ta13	820.5	42.62	3.59	151	0.08	8.6	7.64	66.1	0.49	0.12	—[c)]
Ta14	779.6	26.10	3.57	161	0.14	10.4	2.72	61.4	1.84	1.68	—[c)]
Ta15	775.2	41.42	3.32	190	0.08	7.4	9.18	40.5	11.20	4.02	—[c)]
Ta16	790.5	29.81	3.03	121	0.10	9.6	4.07	25.4	2.10	3.68	—[c)]
Ta17	820.5	34.65	3.56	207[a)]	0.10	—[b)]	—	25.5	1.68	—	—[c)]
Ta18	814.6	31.63	4.31	193	0.14	8.2	4.20	33.1	3.84	3.29	—[c)]
Ta19	779.6	38.34	3.14	174	0.08	8.4	9.18	36.7	8.44	4.27	70.40
Ta20	813.0	28.42	3.20	198	0.11	15.6	4.77	22.8	1.32	6.15	—[c)]
Ta21	779.6	28.27	2.66	234[a)]	0.09	16.1	7.84	25.5	2.43	—	—[c)]
Ta22	775.2	31.46	3.49	148	0.11	7.9	6.16	46.2	7.42	4.60	—[c)]
Ta23	783.3	29.86	4.13	151	0.14	9.0	6.09	20.8	2.80	5.28	—[c)]
Ta24	808.8	27.90	5.02	180	0.18	9.0	4.03	17.8	1.39	4.07	—[c)]
Ta25	783.3	29.97	4.25	217[a)]	0.14	9.3	6.23	16.5	1.73	—	—[c)]
Ta26	790.5	29.87	5.21	181	0.17	9.3	5.43	19.0	1.90	4.75	—[c)]

[a)] The value of r_n exceeds the threshold for valid measurement of $R(0)$ as per Section *Measurements of fracture initiation toughness*;

[b)] Values of w_c and F_c could not be obtained for this specimen since we could not reliably identify the pop-in event.

[c)] In this specimen, the value of ΔF_c exceeded 15% of the value of F_c and therefore we did not consider crack growth to be predominantly stable.

Supplementary References

[1] Monn, M. A. & Kesari, H. A new structure-property connection in the skeletal elements of the marine sponge tethya aurantia that guards against buckling instability. *Scientific Reports* **7**, 1–10 (2017).

[2] Davidge, R. & Tappin, G. The effective surface energy of brittle materials. *Journal of Materials Science* **3**, 165–173 (1968).

[3] Hughes, T. J. *The Finite Element Method: Linear Static and Dynamic Finite Element Analysis* (Courier Corporation, North Chelmsford, Massachusetts, USA, 2012).

[4] Ambrosio, L. & Tortorelli, V. M. *On the Approximation of Free Discontinuity Problems* (Scuola Normale Superiore, Pisa, Italy, 1990).

[5] Ambrosio, L. & Tortorelli, V. M. Approximation of functional depending on jumps by elliptic functional via t-convergence. *Communications on Pure and Applied Mathematics* **43**, 999–1036 (1990).

[6] Francfort, G. A. & Marigo, J.-J. Revisiting brittle fracture as an energy minimization problem. *Journal of the Mechanics and Physics of Solids* **46**, 1319–1342 (1998).

[7] Borden, M. J., Verhoosel, C. V., Scott, M. A., Hughes, T. J. & Landis, C. M. A phase-field description of dynamic brittle fracture. *Computer Methods in Applied Mechanics and Engineering* **217**, 77–95 (2012).

[8] Bourdin, B., Francfort, G. A. & Marigo, J.-J. Numerical experiments in revisited brittle fracture. *Journal of the Mechanics and Physics of Solids* **48**, 797–826 (2000).

[9] Chambolle, A. Addendum to an approximation result for special functions with bounded deformation. j. math. pures appl.(9) 83 (7)(2004) 929–954]: the n-dimensional case. *Journal de Mathématiques Pures et Appliquées* **84**, 137–145 (2005).

[10] Bourdin, B., Francfort, G. A. & Marigo, J.-J. The Variational Approach to Fracture. *Journal of Elasticity* **91**, 5–148 (2008).

[11] Amiri, F., Millán, D., Shen, Y., Rabczuk, T. & Arroyo, M. Phase-field modeling of fracture in linear thin shells. *Theoretical and Applied Fracture Mechanics* **69**, 102–109 (2014).

[12] Miehe, C., Welschinger, F. & Hofacker, M. Thermodynamically consistent phase-field models of fracture: Variational principles and multi-field fe implementations. *International Journal for Numerical Methods in Engineering* **83**, 1273–1311 (2010).

[13] Mesgarnejad, A., Bourdin, B. & Khonsari, M. Validation simulations for the variational approach to fracture. *Computer Methods in Applied Mechanics and Engineering* **290**, 420–437 (2015).

[14] Miehe, C., Hofacker, M. & Welschinger, F. A phase field model for rate-independent crack propagation: Robust algorithmic implementation based on operator splits. *Computer Methods in Applied Mechanics and Engineering* **199**, 2765–2778 (2010).

[15] Wu, T., Carpiuc-Prisacari, A., Poncelet, M. & De Lorenzis, L. Phase-field simulation of interactive mixed-mode fracture tests on cement mortar with full-field displacement boundary conditions. *Engineering Fracture Mechanics* **182**, 658–688 (2017).

[16] Amor, H., Marigo, J.-J. & Maurini, C. Regularized formulation of the variational brittle fracture with unilateral contact: Numerical experiments. *Journal of the Mechanics and Physics of Solids* **57**, 1209–1229 (2009).

[17] Li, T., Marigo, J.-J., Guilbaud, D. & Potapov, S. Gradient damage modeling of brittle fracture in an explicit dynamics context. *International Journal for Numerical Methods in Engineering* **108**, 1381–1405 (2016).

[18] Strobl, M. & Seelig, T. On constitutive assumptions in phase field approaches to brittle fracture. *Procedia Structural Integrity* **2**, 3705–3712 (2016).

[19] Barthelat, F., Tang, H., Zavattieri, P. D., Li, C.-M. & Espinosa, H. D. On the mechanics of mother-of-pearl: a key feature in the material hierarchical structure. *Journal of the Mechanics and Physics of Solids* **55**, 306–337 (2007).

[20] Chen, P. Y., Sheppard, F. A., Curiel, J. M. & McKittrick, J. Fracture mechanisms of bone: A comparative study between antler and bovine femur. *MRS Proceedings* **1132**, 1–6 (2008).

[21] Jackson, A., Vincent, J. & Turner, R. The mechanical design of nacre. *Proceedings of the Royal society of London Series B: Biological Sciences* **234**, 415–440 (1988).

[22] Monn, M. A. & Kesari, H. Enhanced bending failure strain in biological glass fibers due to internal lamellar architecture. *Journal of the Mechanical Behavior of Biomedical Materials* **76**, 69–75 (2017).

[23] Gere, J. M. & Timoshenko, S. P. *Mechanics of Materials*, 351–384 (PWS, Boston, Massachusetts, USA, 1997).

[24] Rho, J. Y., Ashman, R. B. & Turner, C. H. Young's modulus of trabecular and cortical bone material: ultrasonic and microtensile measurements. *Journal of biomechanics* **26**, 111–119 (1993).
